# True Origin of Amide I Shifts Observed in Protein Spectra Obtained with Sum Frequency Generation Spectroscopy

**DOI:** 10.1021/acs.jpclett.3c00391

**Published:** 2023-05-22

**Authors:** Kuo-Yang Chiang, Fumiki Matsumura, Chun-Chieh Yu, Daizong Qi, Yuki Nagata, Mischa Bonn, Konrad Meister

**Affiliations:** † Max Planck Institute for Polymer Research, 55128 Mainz, Germany; ‡ Department of Chemistry and Biochemistry, Boise State University, Boise, Idaho 83725, United States

## Abstract

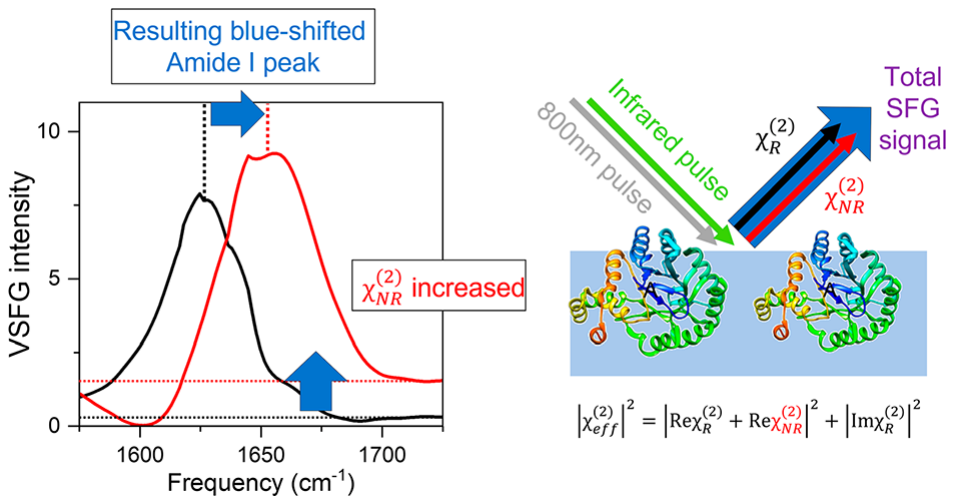

Accurate determination of protein structure at interfaces is critical for understanding protein interactions, which is directly relevant to a molecular-level understanding of interfacial proteins in biology and medicine. Vibrational sum frequency generation (VSFG) spectroscopy is often used for probing the protein amide I mode, which reports protein structures at interfaces. Observed peak shifts are attributed to conformational changes and often form the foundation of hypotheses explaining protein working mechanisms. Here, we investigate structurally diverse proteins using conventional and heterodyne-detected VSFG (HD-VSFG) spectroscopy as a function of solution pH. We reveal that blue-shifts of the amide I peak observed in conventional VSFG spectra upon lowering the pH are governed by the drastic change of the nonresonant contribution. Our results highlight that connecting changes in conventional VSFG spectra to conformational changes of interfacial proteins can be arbitrary, and that HD-VSFG measurements are required to draw unambiguous conclusions about structural changes in biomolecules.

Nature uses proteins as nanomachines to serve as engineers, builders, and destructors of soft and hard biogenic materials.^1−6^ Specialized proteins catalyze reactions, form templates upon crystal growth, and are capable of stabilizing or destroying cellular membranes. The control proteins wield over surfaces is remarkable, and the molecular understanding of their structure and functions at interfaces is key for disciplines as diverse as drug design, biomimetics, or material science.^5,7,8^ Despite the importance of understanding proteins at interfaces, there are currently a limited number of surface-sensitive and label-free techniques available; experimental tools that can accurately determine biomolecular structures at interfaces are highly required.

Vibrational sum frequency generation spectroscopy (VSFG) is a nonlinear optical technique that has shown excellent capabilities to probe interfaces and the structure, orientation, and dynamics of biomolecules with high selectivity.^9^ Conventional VSFG, which provides the SFG intensity (|χ^(2)^|^2^) has often been employed to investigate the proteins’ conformational changes at interfaces such as biological membranes.^10−15^ Analysis of protein structures using VSFG typically relies on probing the amide I backbone vibration. The amide I vibration arises predominately from the C=O stretching vibration of the peptide bond with minor contributions from the out-of-phase C–N stretching vibration, and is sensitive to the protein conformation.^16^ Shifts in the amide I frequency region are routinely assigned to changes in the folding state or conformation of the protein and oftentimes form the basis of novel working mechanisms.^14,15,17,18^ The molecular origin of conformational changes has been explained with altered intermolecular interactions at the interface (e.g., electrostatics, hydrogen bonding, hydrophobic interactions, van der Waals forces) or the modified interfacial interactions with ions and solvent molecules. In addition, environmental factors like temperature, hydrophobicity, cosolutes, and the solution pH are known to strongly affect the structure and properties of proteins.

A significant drawback of the conventional VSFG technique lies in the fact that it provides only |χ^(2)^|^2^ and not the complex χ^(2)^. Yet, the dissipative part of the molecular response, i.e., the equivalent of infrared absorption, is reflected solely by the imaginary part of the complex χ^(2)^ (Imχ^(2)^), and |χ^(2)^|^2^ is typically strongly influenced by the dispersion of the real part of χ^(2)^ (Reχ^(2)^). Furthermore, the phase information is missing in |χ^(2)^|^2^, prohibiting access to the absolute orientation of chemical moieties, which can be inferred from the positive or negative sign of Imχ^(2)^.^19^ Heterodyne-detected VSFG (HD-VSFG) allows us to experimentally determine the detailed shape of Imχ^(2)^ and the phase of χ^(2)^, enabling us to determine the protein structure and water molecules hydrated to the protein more accurately.^20−23^

We investigate the interfacial structures of different proteins at various solution pH levels by performing HD-VSFG measurements in the achiral *s*-SFG, *s*-VIS, and *p*-IR (*ssp*) polarization combination. The conventional VSFG spectra of the proteins showed blue-shifts in the amide I mode peak as the solution pH was lowered, which one might interpret as a change in the protein’s secondary structure from e.g., α-helix to random coil. However, with HD-VSFG, we found that the amide I peak shift in the Imχ^(2)^ spectra, which arises solely from molecular absorption, can actually be red-shifted. We reveal that the blue-shift in the |χ^(2)^|^2^ spectra was caused by changes in the nonresonant signal in the Reχ^(2)^ spectra due to changes of the solution pH. Our analysis demonstrates that the lowering/elevation of the nonresonant signal arises from the changes in the protein’s surface charge induced by the pH change in the solution.

Depending on the ionization state of the charged side chains, proteins can obtain a net positive, net negative, or neutral charge state. This property is directly dependent on the solution pH. The top panel of Figure 1 shows the schematic structure of an antifreeze protein from *Tenebrior Molitor* (TmAFP), a hydrophobin (HstarB), and bovine serum albumin (BSA). The three protein structures contain different secondary structures, with TmAFP being a β-solenoid, HstarB having a globular structure containing helices and β-sheets, and BSA being mostly helical. Apart from their size and structure, the proteins contain different amounts of charged residues and vary in their respective isoelectronic points (IEP). The IEP of a protein describes the pH value at which the overall net charge of the protein is minimal, electrostatic interactions diminish, and other weak forces like hydrogen bonding, hydrophobic interactions, and van der Waals dominate and determine protein structure and stability.

**Figure 1:**
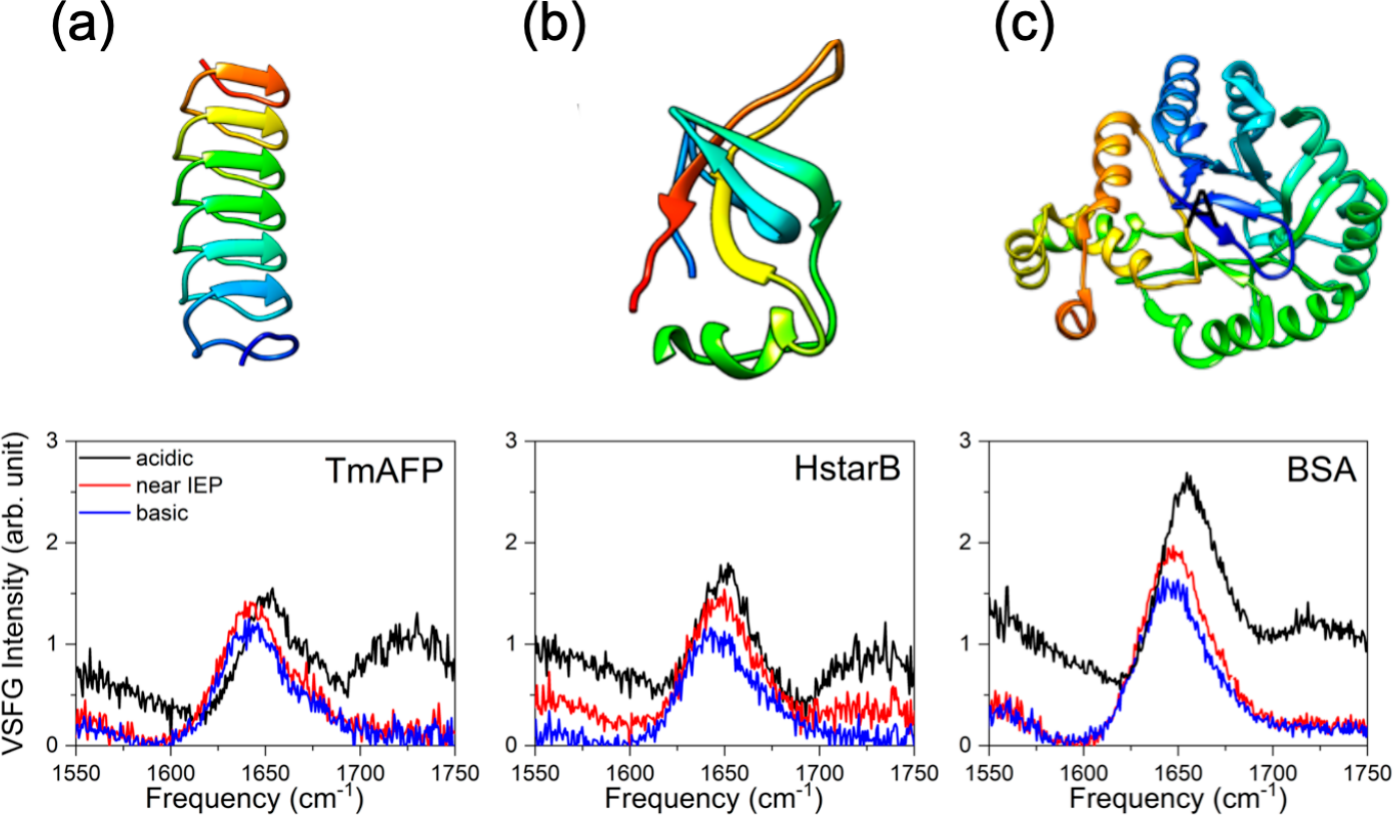
Conventional VSFG spectra of 1g/L TmAFP (a), HStarB (b), and BSA (c) at the deuterated water/air interface for different bulk pDs. Acidic, near IEP, and basic indicate pD ∼3, ∼6, and ∼11, respectively. The ionic strength was held at 300 mM by adding NaCl to the solution containing proteins. Schematic pictures of the bulk protein structures are displayed on the top of the VSFG spectra. Note that the VSFG intensity spectra presented here were obtained directly from the conventional VSFG method.

Figure 1 shows the measured conventional VSFG |χ^(2)^|^2^ spectra at the air/deuterated water (D_2_O) solution of the TmAFP, HstarB, and BSA interface in the frequency region from 1550–1750 cm^–1^, measured in the achiral *ssp* (*s*-SFG, *s*-VIS, *p*-IR) polarization configuration. The spectra were recorded in D_2_O to avoid spectral interference with the H–O–H bending mode of H_2_O. To investigate the pD dependence of these proteins, we conducted VSFG measurements under acidic, near IEP, and basic conditions. The concentrations of the proteins were 1 g/L, and the ionic strength of the solutions was 300 mM by adding NaCl. IEPs of the proteins were estimated to be ∼5.9, ∼ 6.4, and 6.2, respectively. First, we focus on the data of the TmAFP sample shown in Figure 1 (a). Under basic conditions (alkaline, pD = 11, blue line) and close to the IEP (pD = 6, red line), the VSFG spectra show a single, dominant peak centered at ∼1650 cm^–1^. The ∼1650 cm^–1^ peak is assigned to amide I modes, and reflects the β-helical structures for TmAFP,^24^ α-helical structures for HstarB and BSA.^25^ When the pH changes to an acidic pD = 3, a new peak appears at ∼1725 cm^–1^. This peak appears due to the acid–base reaction of the carboxyl group of the amino acid side chain. Since all three proteins contain a C-terminus and a number of negatively charged amino acids, such as aspartic or glutamic acid, we assign the peak at 1725 cm^–1^ to the C=O stretching mode of the protonated carboxylic acid group of the side chains of aspartic and glutamic acid and the C-terminus of the protein. Furthermore, the ∼1650 cm^–1^ amide I peak appears to be blue-shifted to ∼1660 cm^–1^. Such blue-shift of the amide I mode can be interpreted as changes in the protein secondary structures from e.g. α-helix to random coil. As such, the VSFG data indicates that the protein secondary structure is predominantly helical and becomes more random coil upon lowering the pH of the solution at the D_2_O-air interface.

Similar trends can be observed for the other proteins HstarB (Figure 1 (b)) and BSA (Figure 1 (c)). Again, we observe that the main amide I signal centered at ∼1650 cm^–1^ shifts to higher frequencies (∼1660 cm^–1^) when the pD is decreased to acidic conditions, while a slight shift to lower frequencies is observed when the pD is increased. In addition, a new signal at ∼1725 cm^–1^ appears when the pD is decreased. In fact, literature results from various research laboratories reported the same trends.^11,13,26^ It seems that based on the VSFG data, all of the proteins change to a random coil structure at the water–air interface. This is surprising, given that proteins like hydrophobins and TmAFP are known to be pH stable.

A |χ^(2)^|^2^ spectrum contains not only the molecular absorption response (Imχ^(2)^) but also the dispersion response (Reχ^(2)^). A question is whether the blue-shift of the amide I mode in the acid condition arises from the change of the molecular absorption vibration. To address this question, we carried out the HD-VSFG experiments for the same samples in the *ssp* polarization configuration. The top panels of Figure 2 show the Imχ^(2)^ spectra. Under basic conditions and conditions close to the IEP of the proteins, all three Imχ^(2)^ spectra show the strong positive amide I peaks at ∼1640 cm^–1^. When the pH of the solution becomes acidic, a complicated variation of the spectra is revealed. For the TmAFP and HstarB proteins, the amide I peak is red-shifted, in stark contrast to the conventional VSFG data, while for the BSA protein, it is blue-shifted. The blue-shift of the amide I band of the BSA can be assigned to the unfolding of α-helical structures as previously suggested.^25,27,28^ The red-shift of the amide I bands of TmAFP and HstarB is attributed to the orientational or small conformational change in these proteins, given that TmAFP and HstarB are pH stable. To verify that the two data sets are consistent, we calculated the |χ^(2)^|^2^ spectra from the real and imaginary parts obtained in the HD-VSFG experiments (lower panel row in Figure 2), and these data also show a blue-shift of the amide I response under acidic conditions. The spectral positions and shifts under the neutral and alkaline conditions are similar to the conventional VSFG data. However, it is worth pointing out that directly comparing the absolute peak positions and line shapes from our conventional VSFG data and HD-VSFG data is not tangible due to the different system configurations of the SFG measurements.^29,30^

**Figure 2:**
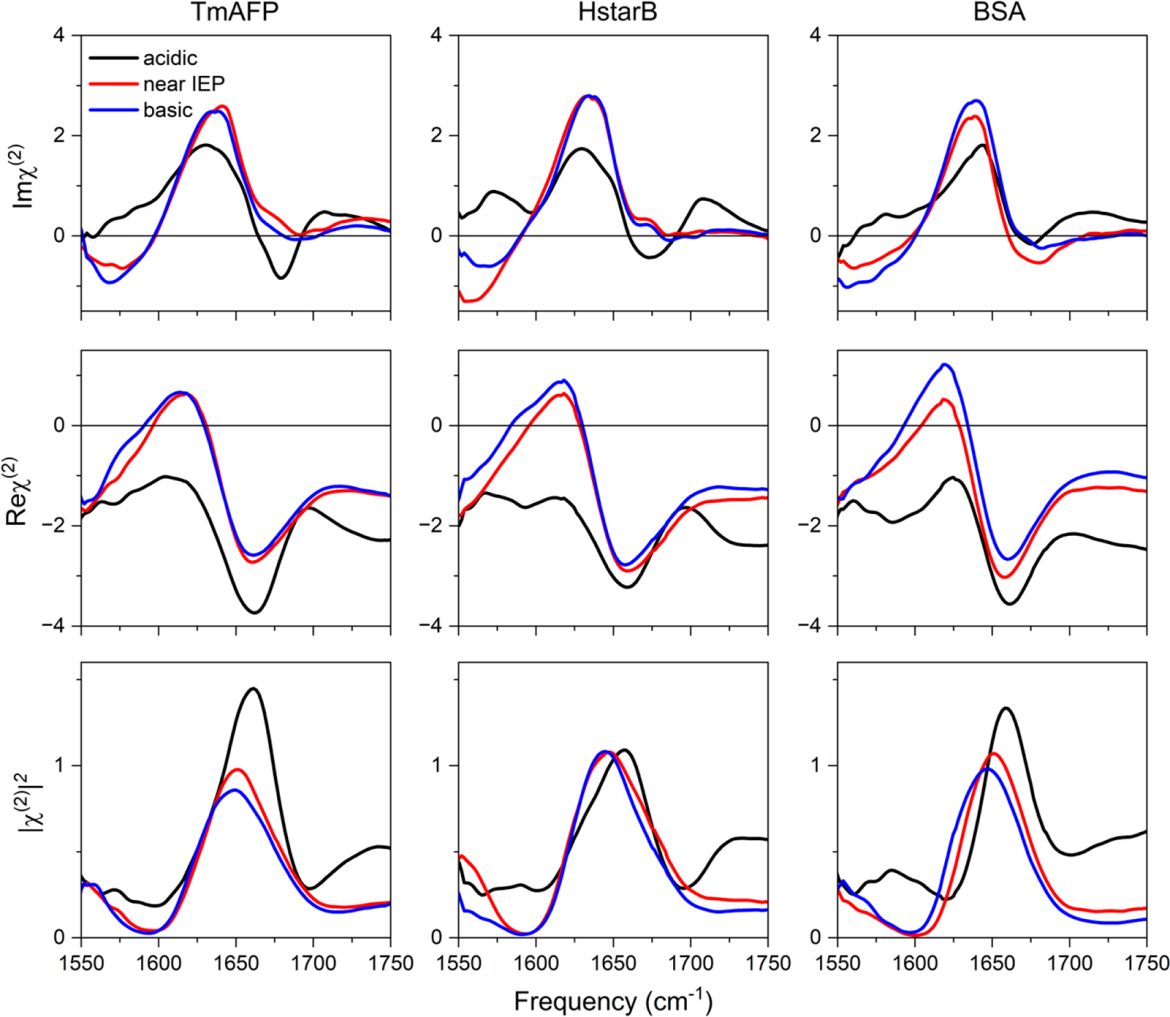
Imaginary and real parts of HD-VSFG spectra (Imχ^(2)^ and Reχ^(2)^, respectively) of 1g/L TmAFP, HStarB, and BSA at the deuterated water/air interface for different bulk pDs. Acidic, near IEP, and basic indicate pD ∼3, ∼6, and ∼11, respectively. The bottom panel shows the constructed |χ^(2)^|^2^ to compare with the conventional VSFG spectra shown in Figure 1. Note that the presented constructed |χ^(2)^|^2^ is calculated via |χ^(2)^|^2^ = |Reχ^(2)^|^2^ + |Imχ^(2)^ |^2^. All HD-VSFG spectra were measured in deuterated water with an ionic strength of 300 mM NaCl.

What is the origin of the seemingly contradicting results regarding the central amide I frequency from the SFG intensity and Imχ^(2)^ spectra? To explain this, we focus on the Reχ^(2)^ spectra shown in the center row panels of Figure 2. One can see that the baseline (nonresonant component) is lowered in all three Reχ^(2)^ spectra. This lowering of the baseline has a significant impact on the |χ^(2)^|^2^ data, because Reχ^(2)^ is amplified in the |χ^(2)^|^2^ data.

A question arising here is why the nonresonant part in the Reχ^(2)^ spectra varies with pH. Two scenarios are possible. One scenario is that elevation/lowering of the pH changes the nonresonant contribution directly, while the other scenario is that the variation of the charges on protein induced by the pH change alters the nonresonant contribution, i.e., indirectly. To examine the first scenario, we measured HD-SFG spectra of the HCl and NaOH aqueous solutions at the water–air interface in the 2000–2200 cm^–1^ region. We chose the frequency region slightly higher than the amide I region, to see the nonresonant contribution solely by avoiding the resonant contribution of the vibrational modes, such as the bending mode of water. The Reχ^(2)^ data is displayed in Figure 3 (a). This shows that the change in the nonresonant contribution is negligible. Thus, the first scenario can be ruled out.

**Figure 3:**
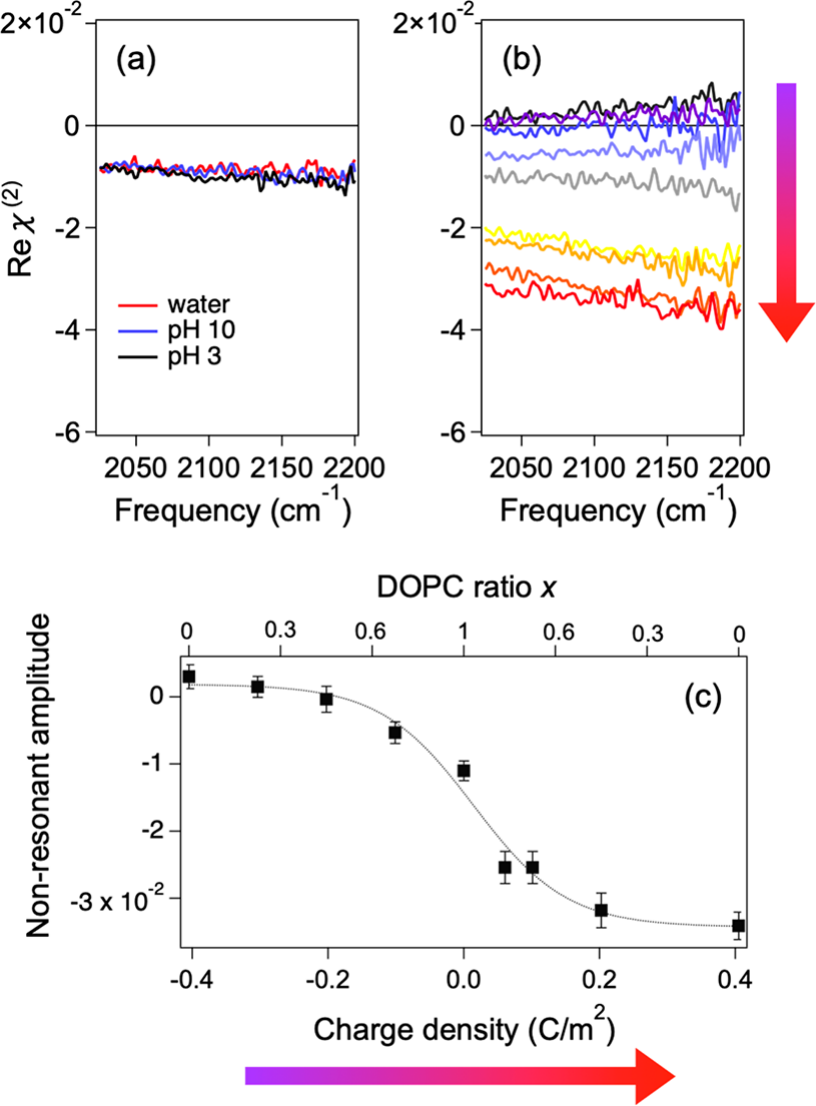
(a) Real part of HD-VSFG spectra (Reχ^(2)^) at the water–air, HCl solution–air, and NaOH solution–air interfaces. (b) Reχ^(2)^ spectra at the water–lipid interfaces. The composition of the lipid is *x*DOPC+(1 – *x*)DPPG/DPTAP. The surface charge is computed from the charge of the lipid. (c) Amplitude of the nonresonant part versus surface charge. The nonresonant amplitude was obtained by averaging the amplitude in panel (b) from 2000 to 2200 cm^–1^. Error bar shows standard deviation. The line is a sigmoidal fit to guide the eye. The arrows with color gradient in (b) and (c) indicate the charge densities.

To examine the second scenario, we measured the HD-VSFG spectra at the water–lipid interface. By mixing charge-neutral DOPC lipid with positively charged DPTAP (negatively charged DPPG) lipid with various ratios, we controlled the absolute surface charge.^31,32^ The data is shown in Figure 3 (b), while the nonresonant contribution vs surface charge is summarized in Figure 3 (c) and is consistent with previous studies.^33,34^ It is clear that the nonresonant contribution varies drastically with varying surface charge, consistent with the observation for the protein samples. Our results manifest that the nonresonant variation arises from the change of the surface charge induced by the pH change.

The profile of the nonresonant Reχ^(2)^ contribution vs surface charge provides information on the isoelectric point at the interface. The unchanged nonresonant contribution in the protein signal under basic and pH = 6 (near IEP) conditions indicates that, near the bulk IEP, the protein at the surface retains its negative charge, rather than a net zero charge. This means that the isoelectric point at the aqueous interface tends to be lower than that in the bulk. This notion is consistent with a previous study reporting that the proteins at interfaces have an isoelectric point lower by 1 at the interface than in the bulk.^22^

To further understand how the nonresonant contribution affects the peak position, we calculate the constructed |χ^(2)^|^2^ spectra by offsetting the nonresonant contribution in Reχ^(2)^ of near IEP HD-VSFG data for three different proteins. By doing this, we examine the effect of different nonresonant offsets in |χ^(2)^|^2^ spectra. We use the near IEP HD-VSFG data to calculate the constructed |χ^(2)^|^2^ spectra with |χ^(2)^|^2^ = |Reχ_*R*_^(2)^ + χ_*NR*_^(2)^ + Δχ_*NR*_^(2)^|^2^ + |Imχ_*R*_^(2)^|^2^, where χ^(2)^ = χ_*R*_^(2)^ + χ_*NR*_^(2)^ and Imχ_*NR*_^(2)^ = Im(Δχ_*NR*_^(2)^) = 0. We set Δχ_*NR*_^(2)^ = ± 0.018 since the maximum change of the nonresonant term is estimated to be ∼ ± 0.018 derived from Figure 3. These results are shown in Figure 4. Under conditions where Δχ_*NR*_^(2)^ = +0.018, the total nonresonant contribution is almost zero, leading to the typical Lorentzian line shape. As the nonresonant contribution increases negatively in the Reχ^(2)^ signal, the peak position of the amide I mode can blue-shift ∼30 cm^–1^ in the |χ^(2)^|^2^ spectra. Such a signature often appears when Reχ^(2)^ spectra cross the zero line. We highlight that the peak shift caused by the nonresonant change can mask the spectral responses of protein structural changes in the Imχ^(2)^. This observation likely explains why there are ∼10 cm^–1^ blue-shifts of the amide I peaks for the TmAFP and HstarB proteins in the conventional VSFG data, while there are both 5 cm^–1^ red-shifts of the peaks in the Imχ^(2)^ spectra.

**Figure 4:**
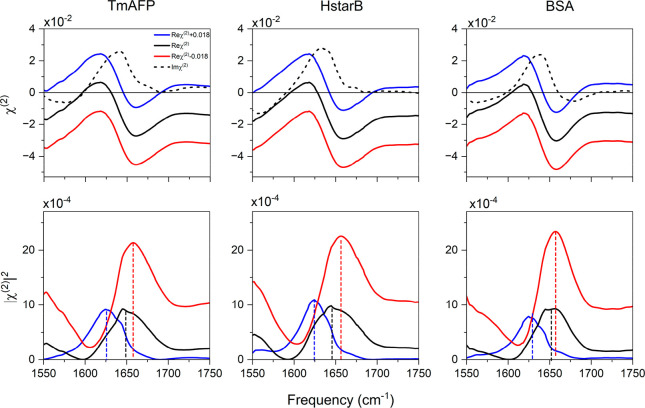
Effects of nonresonant contributions χ_*NR*_^(2)^ on the constructed |χ^(2)^|^2^ spectra for TmAFP, HstarB, and BSA. The offsets Δχ_*NR*_^(2)^ of the nonresonant term were estimated to be ∼ ± 0.018 from Figure 3. The solid and dashed black lines in the upper panel are the original real and imaginary parts of near IEP χ^(2)^ spectra from Figure 2. The blue and red lines represent the positive and negative enhancements of the nonresonance signal. The dashed lines in the lower panels indicate the middle points of the peak fwhm in the |χ^(2)^|^2^ spectra.

We report a clear blue-shift in the amide I peak for conventional VSFG |χ^(2)^|^2^ data for different model proteins, suggesting changes in the protein’s respective structure or orientation. In contrast to the conventional VSFG data, HD-VSFG data reveal that the amide I peak frequency in the Imχ^(2)^ data can be rather red-shifted. This seeming contradiction can be understood by noting that in the|χ^(2)^|^2^ signal, there is strong interference with the nonresonant signal in the real part, as observed in HD-VSFG measurements. Clearly, unravelling structural changes of proteins at the interface cannot be studied by solely observing peak-shifts in conventional VSFG |χ^(2)^|^2^ data. We unveiled that the nonresonant signal changes substantially due to the charge variation of surface protein molecules induced by the change in the solution pH.

Here, we would like to review how the peak frequency was obtained from the conventional VSFG data and point out the possible limitations. A typical route to obtain the peak frequency uses the fit of the Lorentzian lineshapes to the |χ^(2)^|^2^ spectra. However, the peak is often not Lorentzian, leading to the error of the fitting. In fact, the fit of the Lorentzian shapes in the Imχ^(2)^ and Reχ^(2)^ data demonstrates that the Lorentzian lineshapes cannot be used to reproduce the Reχ^(2)^ spectra, in particular (see Figure S1 in Supporting Information). Furthermore, the unclear sign of the resonant contribution gives rise to the uncertainty of the peak frequency (see Figure S2 in Supporting Information). More advanced studies use the VSFG spectra calculation to connect the protein structure and the conventional VSFG signal.^11,12,35^ However, in the simulation, one often assumes that protein is not aggregated.^12^ Furthermore, the nonresonant signal is not predicted from the simulation and thus is assumed,^36^ providing the uncertainty in the agreement. The uncertainty increases when the nonresonant background is changed, as is discussed in Figure 3. When the nonresonant signal is small, we can skip some limitations, but this is not always the case, as is discussed in the current manuscript. As such, HD-VSFG signals of proteins provide unique and critical platforms to compare the simulated and experimental data.

We emphasize that the use of HD-VSFG measurements is crucial for accurately interpreting amide I spectral shifts, which reflects protein structures and conformational changes at the interface. We further conjecture that HD-VSFG measurements can provide valuable new information to investigations of membrane protein folding processes that are influenced by membrane parameters such as headgroup charge as they can detect both potential red-shifts of amide I peak in Imχ^(2)^ spectra and the blue-shifts in |χ^(2)^|^2^ spectrum, which may not have been detected in conventional VSFG studies.

## Supporting Information Available

Sample preparation of protein solutions and DOPC/DPTAP and DOPC/DPPG mixtures surface monolayer, details of conventional VSFG and HD-VSFG experimental setup, fitting data of the HD-VSFG measurement, circular dichroism spectroscopy measurements, surface tension measurements, and BAM measurements (PDF)
