# Supplementary material for: True Origin of Amide I Shifts Observed in Protein Spectra Obtained with Sum Frequency Generation Spectroscopy
Source: J Phys Chem Lett. 2023 May 22;14(21):4949–54. doi: 10.1021/acs.jpclett.3c00391 (PMC10240526; doi:10.1021/acs.jpclett.3c00391)
Supplement: Supplementary file 1 — jz3c00391_si_001.pdf [file jz3c00391_si_001.pdf]

Supporting Information for

True Origin of Amide I Shifts Observed in Protein Spectra Obtained with Sum Frequency Generation Spectroscopy

Kuo-Yang Chiang[1§], Fumiki Matsumura[1§], Chun-Chieh Yu[1], Daizong Qi[1], Yuki Nagata[1], Mischa Bonn[1], Konrad Meister[1,2]*

[1]Max Planck Institute for Polymer Research, 55128 Mainz, Germany

[2]Department of Chemistry and Biochemistry, Boise State University, Boise, Idaho 83725, USA

*Correspondence: meisterk@mpip-mainz.mpg.de

Conventional Vibrational Sum-Frequency Generation (VSFG) measurement

For the conventional SFG setup, the measurements were performed on a femtosecond Ti: Sapphire amplified laser system (Coherent Libra, ~800 nm, ~50 fs, 1 kHz) with 5 W output power. We used 2 W to pump an optical parametric amplifier (TOPAS, light conversion) with a noncollinear DFG stage to generate the broadband IR pulse. Another 1 W of the laser output was passed through an etalon to generate the narrowband visible pulse (~20 cm^{-1}). The visible and IR beams overlapped spatially and temporally at the sample surface with their incident angles of 45 and 41 degrees with respect to the surface normal, respectively. Subsequently, the SFG signal is dispersed in a spectrometer and detected by an EMCCD camera. The power of visible and IR beams are 13 μJ and 5 μJ per pulse, respectively, at the sample position.

Heterodyne-detected Vibrational Sum-Frequency Generation (HD-VSFG) measurement

For heterodyne-detected VSFG measurements, the experimental setup is described in detail elsewhere[1]. Briefly, a part of the power from a 1-kHz femtosecond Ti: Sapphire laser system (Spectra-Physics) was used to generate a narrowband visible pulse (bandwidth ~14 cm^{-1} at central wavelength 800 nm) by a grating-based pulse shaper. The rest of the power was used to pump the commercial optical parametric amplifier (TOPAS SHBS-400, Spectra-Physics) system with a silver gallium disulfide ($AgGaS_2$) crystal for the generation of broadband mid-infrared pulse (bandwidth ~180 cm^{-1} at center frequency 1650 cm^{-1}). The visible (ω_1) and infrared (ω_2) pulses were first focused on a 20-μm-thick *y*-cut quartz plate to generate the local oscillation (LO) signal. The ω_1, ω_2, and LO pulses were focused again on the sample surface with all the incident angles of 45 degrees. A phase modulator was placed before the sample to delay the LO pulse relative to ω_1 and ω_2 pulses by ~1.3 ps. All the heterodyne measurements are performed at the *ssp* polarization combination, where *ssp* denotes *s*-polarized SFG, *s*-polarized visible, and *p*-polarized IR beams. The SFG signal from the sample interfered with the LO beam, generating the interference fringe. The interference fringe was detected by a liquid nitrogen cooled CCD Camera (Princeton Instruction, PyLoN®). The power of visible and IR beams are 10 μJ and 2 μJ per pulse, respectively, at the sample position.

Fitting Process for HD-SFG Spectra

We performed the spectral fitting for the HD-SFG spectra (Im$\chi^{(2)}_{\text{eff}}$ and Re$\chi^{(2)}_{\text{eff}}$) in Figure 2 via

$$\chi^{(2)}_{\text{eff}}(\omega) = A_{\text{NR}} e^{i\phi_{\text{NR}}} + \sum_n \frac{A_n}{\omega - \omega_n + i\Gamma_n}, \tag{S1}$$

where A_{NR} and ϕ_{NR} are the amplitude and phase of the non-resonant contribution. ω_n, A_n, and Γ_n are the characteristic frequency, amplitude, and linewidth of resonant signal, respectively. The obtained parameters are summarized in Table S1.

Table S1. The fitting parameters of the HD-SFG spectra. The error of the frequency is ~5 cm^{-1}.

	TmAFP			HstarB			BSA		
	Acidic	nearIEP	Basic	Acidic	nearIEP	Basic	Acidic	nearIEP	Basic
A_{NR}	0.019	0.0125	0.0115	0.019	0.0145	0.009	0.215	0.0125	0.009
ϕ_{NR}	180°	180°	180°	180°	180°	180°	180°	180°	180°
A_1	-	0.02	0.05	-	-	-	0.08	0.02	0.1
ω_1	-	1554	1554	-	-	-	1554	1554	1554
Γ_1	-	20	20	-	-	-	20	20	20
A_2	-	0.165	0.17	-0.075	0.37	0.15	-0.072	0.175	0.17
ω_2	-	1582	1580	1573	1558	1572	1595	1576	1580
Γ_2	-	18	18	11	25	18	45	23	20
A_3	-0.51	-0.56	-0.625	-0.41	-0.62	-0.72	-0.42	-0.53	-0.6
ω_3	1634	1637	1636	1631	1634	1637	1643	1638	1638
Γ_3	25	20	22	22	20	22	20	18	18
A_4	0.245	0.14	0.184	0.17	0.09	0.25	0.165	0.3	0.184
ω_4	1675	1690	1675	1672	1675	1668	1672	1676	1672
Γ_4	16	26	26	18	26	30	16	26	26
A_5	-0.13	-0.2	-0.07	-0.135	-	-0.01	-0.14	-0.14	-
ω_5	1715	1730	1735	1713	-	1735	1715	1715	-
Γ_5	30	50	40	20	-	40	30	40	-

Difficulty in determining the peak frequency from the homodyne data

We would like to note that this procedure has two major problems which prevent obtaining the correct resonant frequency.

1. For the fit, one typically uses the Lorentzian shape, but protein vibrational modes cannot be well described by the Lorentzian model.

2. The sign of the resonant signal is not known, causing an error of the peak frequency estimation.

To show that the Lorentzian shape is not sufficient to capture the spectral shape (point 1), we performed Lorentzian fits to the real and imaginary part spectra. The data are displayed in Figure S1. The data shows that the real part spectrum is not captured by the Lorentzian model, exhibiting the limitation of the Lorentzian model for describing protein vibrational modes. Since the spectra cannot be captured by the Lorentzian model, the fit using the Lorentzian shapes will inevitably lead to the errors of the peak frequency.

To estimate how much a peak is changed when the sign of the resonant part is not determined, we constructed $|\chi^{(2)}|^2$ spectra when $\chi^{(2)} = \chi_R^{(2)} + \chi_{NR}^{(2)}$ and $\chi^{(2)} = -\chi_R^{(2)} + \chi_{NR}^{(2)}$. This data is shown in Figure S2. When flipping the sign of the $\chi_R^{(2)}$, the position of the peak can shift ~12 cm^{-1}. This clearly indicates that the sign of the $\chi_R^{(2)}$ has a significant effect on the peak position in $|\chi^{(2)}|^2$ spectra. Thus, without knowing the sign of the resonant signal, determination of the peak position through fitting can be challenging.

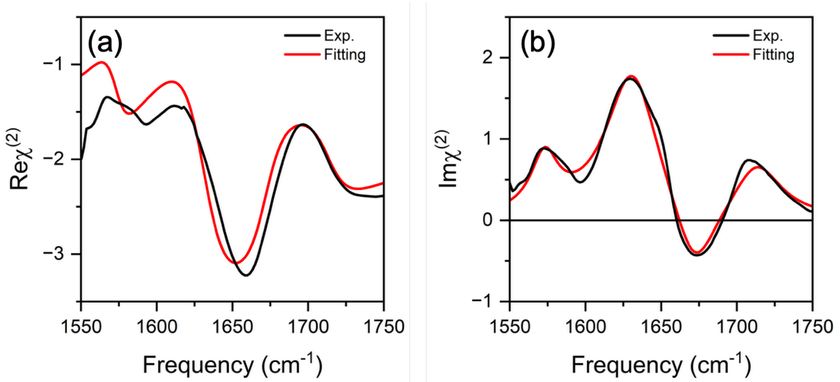

Figure S1. Fitting of the experimental HD-VSFG data (HstarB under acid condition in Figure 2) with Lorentzian functions. The result of the (a) real part and (b) imaginary part. Black solid lines represent the experimental result, while red solid lines denote the fitting result.

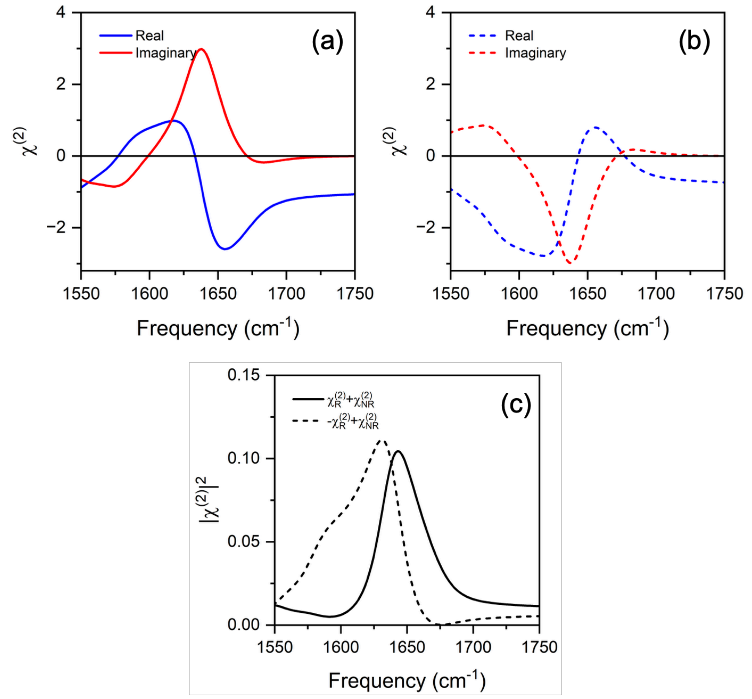

Figure S2. The effect of the sign of the resonant signal $\chi_R^{(2)}$ on the constructed $\left|\chi^{(2)}\right|^2$ spectra. Real and imaginary parts of the $\chi_R^{(2)}$ when the peak at ~1640 cm^{-1} in the imaginary part has a positive sign (a), and when the peak at ~1640 cm^{-1} in the imaginary part has a negative sign (b). (c) Constructed $\left|\chi^{(2)}\right|^2$ spectra calculated from the data shown in (a) and (b). We used the fitting data of BSA under basic condition for extracting $\chi_R^{(2)}$ and $\chi_{NR}^{(2)}$.

CD spectroscopy measurement

CD spectra were recorded at a 1 nm interval from 260 to 180 nm using a Jasco J-1500 spectrometer. CD measurements were performed in a rectangular cell with an optical path of 0.1 cm and at a concentration of 1 mg/mL in H_2O at 22 °C. The CD spectra do not show significant changes under the different pD conditions, which indicates that the protein structures are not or are only slightly altered.

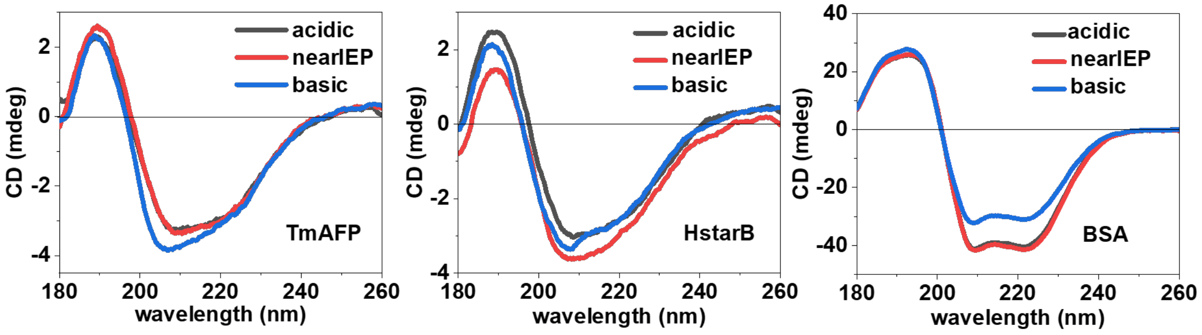

Figure S3. CD Spectra of the proteins (TmAFP, HstatB and BSA) at three different pD conditions (acidic, near IEP, and basic) at a concentration of 1 mg/mL in H_2O at 22 °C.

Surface Tension measurement

Surface tension measurements were performed in a custom-made Teflon trough filled with 5 mL of aqueous sample solutions at room temperature (22 ± 1 °C) using a DeltaPi tensiometer (KBN 315 Sensor Head, Kibron Inc.).

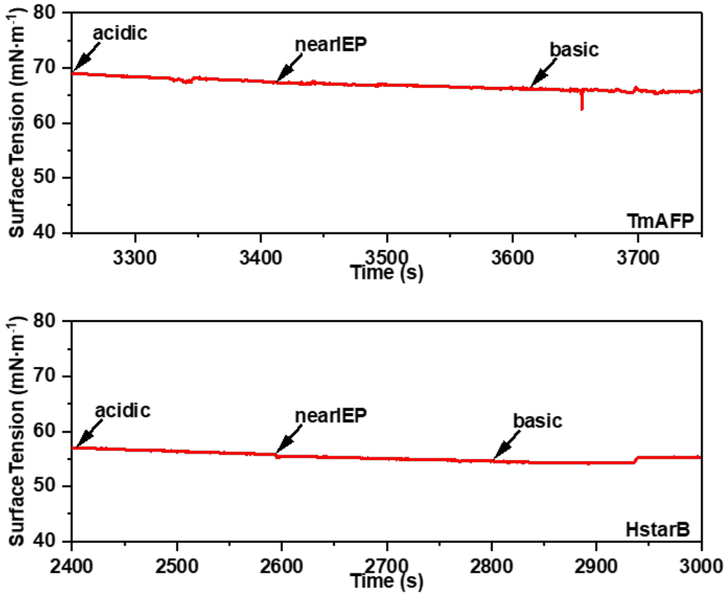

Figure S4. Surface tensions of 1g/L TmAFP and HstarB at the deuterated water/air interface for different bulk pDs.

BAM measurement

BAM image measurements were performed using a commercial setup (Accurion, EP3 BAM).

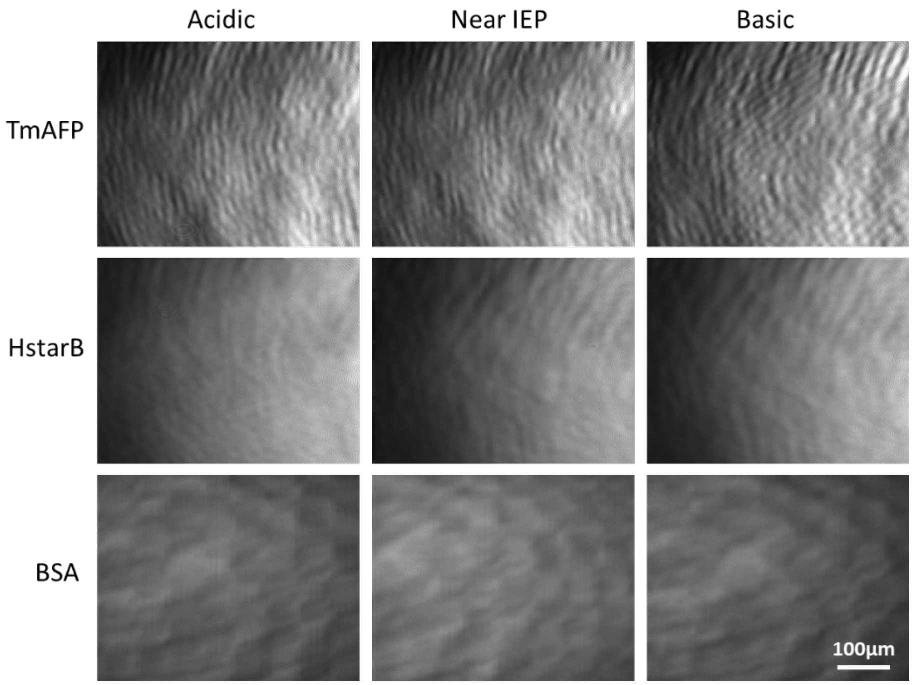

Figure S5. The BAM images of the proteins (TmAFP, HstatB and BSA) at three different pD conditions (acidic, near IEP, and basic).

Sample preparation

Proteins

TmAFP was purified from *Tenebrio Molitor* using ice affinity purification as described elsewhere [4]. Hstar B (BASF) and BSA (Sigma Aldrich) were used without further purification. The IEPs of the TmAFP, Hstar B, and BSA were estimated to be ~5.9, ~6.4, and 6.2, respectively. Experiments under acidic conditions were performed at a solution pD of ~3 and experiment under basic conditions were performed at a solution pD of ~11. The SFG measurements of the proteins were performed under N_2 purging to avoid the absorption by water vapor. To prevent the heat accumulation,[5] the trough was rotated during the measurement. The prepared samples were equilibrated at least for 15 minutes.

DOPC/DPTAP and DOPC/DPPG mixtures

We diluted chloroform solutions of DOPC and DPTAP (Avanti Polar Lipids) with chloroform (Acros Organics, 99.8 %) to make a concentration of 0.43 mM solution. We dissolved chloride salt of DPPG (Avanti Polar Lipids) into the mixture of 10 % methanol (VWR chemicals, 99.8 %) and 90 % chloroform (Acros Organics, 99.8 %) at a concentration of 0.43 mM. The DOPC/DPTAP and DOPC/DPPG samples were prepared by mixing the pure solutions of DOPC, DPTAP, and DPPG at different ratios. The 20 mL H_2O was poured into a Teflon trough with an 8.0 cm diameter. To avoid the distortion of lipid monolayer from heat accumulation[5], the trough was rotated during the measurement. We deposited 50 μL DOPC/DPTAP and DOPC/DPPG mixtures using a click syringe. The prepared samples were equilibrated at least for 15 minutes. The mean molecular area of DOPC/DPTAP(DPPG) mixtures were estimated to be ~40 Å^2. We calculated the surface charge by linear combination of DOPC/DPTAP and DOPC/DPPG ratio, with an assumption that pure DPTAP and DPPG has surface charge of 0.4 C/m^2 and -0.4 C/m^2, respectively. [6]

References

(1) Chiang, K. Y.; Seki, T.; Yu, C. C.; Ohto, T.; Hunger, J.; Bonn, M.; Nagata, Y. The Dielectric Function Profile across the Water Interface through Surface-Specific Vibrational Spectroscopy and Simulations. *Proc. Natl. Acad. Sci. U. S. A.* **2022**, *119*, e2204156119.

(2) Shen, Y. R. Phase-Sensitive Sum-Frequency Spectroscopy. *Annu. Rev. Phys. Chem.* **2013**, *64*, 129–150.

(3) Yu, X.; Chiang, K.-Y.; Yu, C.-C.; Bonn, M.; Nagata, Y. On the Fresnel Factor Correction of Sum-Frequency Generation Spectra of Interfacial Water. *J. Chem. Phys.* **2023**, *158*, 044701.

(4) Adar, C.; Sirotinskaya, V.; Bar Dolev, M.; Friehmann, T.; Braslavsky, I. Falling Water Ice Affinity Purification of Ice-Binding Proteins. *Sci. Rep.* **2018**, *8*, 11046.

(5) Backus, E. H. G.; Bonn, D.; Cantin, S.; Roke, S.; Bonn, M. Laser-Heating-Induced Displacement of Surfactants on the Water Surface. *J. Phys. Chem. B* **2012**, *116*, 2703–2712.

(6) Dreier, L. B.; Nagata, Y.; Lutz, H.; Gonella, G.; Hunger, J.; Backus, E. H. G.; Bonn, M. Saturation of Charge-Induced Water Alignment at Model Membrane Surfaces. *Sci. Adv.* **2018**, *4*, eaap7415.
